# A Quantitative Text Analysis of the 8050 Problem and Stratified Support in the Japanese Diet

**DOI:** 10.7759/cureus.103557

**Published:** 2026-02-13

**Authors:** Takao Sakai

**Affiliations:** 1 Orthopedics, Nagoya Kyoritsu Byoin, Nagoya, JPN

**Keywords:** 8050 problem, hikikomori, japanese health policy, natural language processing (nlp), quantitative text analysis, social determinants of health (sdoh)

## Abstract

Introduction

The "8050 problem," referring to households where elderly parents in their 80s support socially withdrawn (*hikikomori*) children in their 50s, presents a critical public health challenge in Japan. To address such complex social isolation, the Japanese government introduced the "Stratified Support System" in 2021. However, there is a concern that bureaucratic framing may obscure the clinical urgency of the issue. This study employs full-population text analysis to objectively quantify the semantic shift in Japanese legislative discourse regarding the 8050 problem, specifically determining whether the 2020 Social Welfare Act amendment drove a transition from clinical terminology to administrative procedural framing.

Materials and methods

I conducted a comprehensive quantitative text analysis of all minutes from the Japanese National Diet (both the House of Representatives and House of Councillors) over an 11-year period (January 1, 2015, to December 31, 2025). Utilizing the National Diet Library Application Programming Interface (NDL API) to eliminate selection bias, I targeted the entire population of parliamentary statements in Japan. I employed Python-based (Python Software Foundation, Wilmington, DE) natural language processing (NLP) with the morphological analyzer "Janome." I performed a time-series analysis of keyword frequency and co-occurrence network analysis to visualize the semantic structure of policy debates, ensuring their high reproducibility.

Results

Time-series analysis revealed a significant paradigm shift: mentions of "hikikomori" (clinical reality) peaked in 2019 but diminished significantly after 2020. Conversely, mentions of "Stratified Support" (administrative logic) surged after the 2020 Social Welfare Act amendment. The co-occurrence analysis of the new system's discourse demonstrated a dominance of procedural terms such as "cooperation," "consultation," and "system." Notably, outcome-oriented terms related to clinical recovery or the direct resolution of isolation were scarce compared to administrative process terminology.

Conclusions

Legislative discourse on social withdrawal has shifted from clinical realities to administrative coordination. This "depersonalization" of policy language may risk prioritizing institutional maintenance over the direct resolution of social determinants of health (SDOH). While administrative integration is necessary, the findings suggest a potential disconnect between policy frameworks and the lived experiences of vulnerable populations. API-based full-text analysis serves as a vital objective tool for monitoring structural shifts in public health policy.

## Introduction

The issue of "hikikomori," once regarded primarily as a problem among younger generations, has become increasingly serious as the affected population has aged. Specifically, there is a growing trend in which individuals who have withdrawn from society for long periods reach middle or late adulthood (in their 50s) while continuing to be financially and emotionally supported by their elderly parents (in their 80s). This fixed household structure is commonly referred to as the "8050 problem." The need for caregiving due to parental aging or the loss of a parent poses a fundamental threat to household stability, carrying the risk of family collapse. The urgent issue of the "8050 problem" in Japanese public health encompasses complex challenges such as financial hardship, caregiving, mental health, and social isolation from the community [1].

Japan's conventional social welfare system has been structured through "vertical administration," which divides services according to attributes such as the elderly, people with disabilities, children, and those experiencing financial distress. What further exacerbates this situation is the application-based principle, *Shinsei Shugi*, that lies at the core of Japan's welfare system and is also a key principle of the Japanese child welfare system. This principle, which essentially does not support those who do not actively seek help, functions as a virtually fatal barrier for 8050 households that are incapable of asking for help themselves. Even if individuals or families muster the courage to visit a government office, the complex issues they face are often dismissed as "not our department" owing to the compartmentalized nature of administrative organizations and are subject to bureaucratic shuffling between departments. Consequently, those in need become disillusioned and lose trust in the authorities, leading them to withdraw further from society and shut themselves in once more. Thus, the problems remain hidden and intertwined within the closed-off space of the home, forming a mechanism whereby issues go undetected until it is too late, a cycle of concealment and increasing complexity.

The current reliance on application-based models creates a structural mismatch between the welfare system and households experiencing social isolation. To break this deadlock, it is essential to move away from the traditional model, which relies on the user's "help-seeking ability," and move toward an "outreach-oriented model," in which the authorities take proactive involvement [2]. Shifting the burden of initiating support from the "vulnerable individual" to the "support system" itself is the key to dismantling the aforementioned "mechanism of concealment and complexity."

To fundamentally resolve these structural defects, the government has promoted the realization of a "regional symbiotic society," moving toward a more comprehensive support system. As a concrete and practical measure of this shift, the Multilayered Support System Development Project was launched in April 2021. The groundbreaking aspect of this initiative lies in the relaxation of restrictions on the use of national subsidies, allowing budgets that previously belonged to separate fields, such as the elderly, disability, children, and those experiencing livelihood difficulties, to be managed collectively. While building on existing consultation and support frameworks, this project calls for the integrated and multilayered provision of three new functions: the first is a consultation support project without refusal, which offers help regardless of personal attributes. The second is a participation support project that restores individual dignity through initiatives such as employment assistance and the creation of community spaces. The third is a community-building support project that uncovers latent needs through outreach and fosters mutual aid among residents. By organically connecting these functions, horizontal cooperation among previously fragmented support institutions is promoted, and a comprehensive care system is expected to function effectively.

However, the increasing sophistication of administrative systems aimed at encompassing every issue brings new risks. Eliminating arbitrariness and grasping the structure of debates within the vast proceedings of the National Diet were difficult with conventional manual analyses. However, in the field of political science, Catalinac (2016) demonstrated, using a quantitative text analysis of parliamentary records, that Japanese politicians' concerns structurally shifted from favor-seeking to policy-oriented approaches [3].

In this study, a comprehensive quantitative text analysis was conducted on all proceedings of both houses of the National Diet over the past 10 years, utilizing the National Diet Library's Diet Proceedings Search System Application Programming Interface (API) (NDL API). Specifically, natural language processing (NLP) techniques were employed to extract statements related to the 8050 issue and multilayered support from vast text data. Furthermore, by applying morphological analysis to break down and quantify the content of these statements on a word-by-word basis, this study objectively captures the vocabulary used in the discussions without relying on any particular political standpoint or the subjectivity of researchers. This study aims to quantitatively examine changes in the Japanese National Diet discourse regarding the 8050 problem by comparing the frequency and semantic structure of clinical (e.g., *hikikomori*) and administrative (e.g., Stratified Support System) terminology from 2015 to 2025 using full-population, NLP-based text analysis.

## Materials and methods

Data collection and selection

In this study, to quantitatively grasp the transitions of policy debates in the Japanese Diet and the linguistic characteristics in the process of institutionalizing social issues, speech data were collected using the "Minutes Search System API (NDL API)" provided by the National Diet Library [4]. The analysis period covered 11 years, from January 1, 2015, to December 31, 2025, targeting speeches made in plenary sessions and all committees of the House of Representatives and the House of Councillors. In accordance with the NDL's API Terms of Service, the data were accessed for noncommercial research purposes, which do not require specific copyright permission.

For the analysis, two contrasting conceptual categories were set to examine the gap between "issues" in society and "institutions" as solutions to those issues. First, as an indicator of the clinical reality that becomes apparent as a social problem, "hikikomori," often discussed in the context of issues such as the 8050 problem, was selected as a search keyword. Second, as an indicator of comprehensive administrative measures addressing the issue (administrative logic), "Stratified Support," established by the 2020 amendment to the Social Welfare Act, and its official name, "Project for Developing a Stratified Support System," were chosen. To ensure the full replicability of the text-mining process, the exact API parameters, preprocessing steps, token filtering rules, and stop-word lists used in this study are detailed in the Appendices.

Analytical approach

The following analyses were conducted using a single prompt.

First, a "time-series analysis" was performed. Here, the number of annual occurrences of speeches containing each selected keyword was comprehensively totaled to visualize when each concept attracted attention within the Diet and how it transitioned over time. In particular, the analysis focused on the correlation between the policy turning point marked by the 2020 amendment to the Social Welfare Act and changes in the keyword frequency.

Second, a "semantic structure analysis" was conducted. For this, the full text of speeches related to the administrative solution "Project for Developing a Stratified Support System" was analyzed using Janome, a Japanese morphological analysis library, to segment the text and perform part-of-speech (POS) tagging. In the analysis, function words such as particles and auxiliary verbs were excluded, and only nouns with semantic content were extracted for term frequency calculations. Furthermore, to clarify the associations between words in context, a co-occurrence analysis was conducted by tallying word combinations that appeared together in the same speech. This allowed for the examination of the main points of contention in institutional design and the logical structure used by the administration to address the issues.

## Results

**Figure 1:**
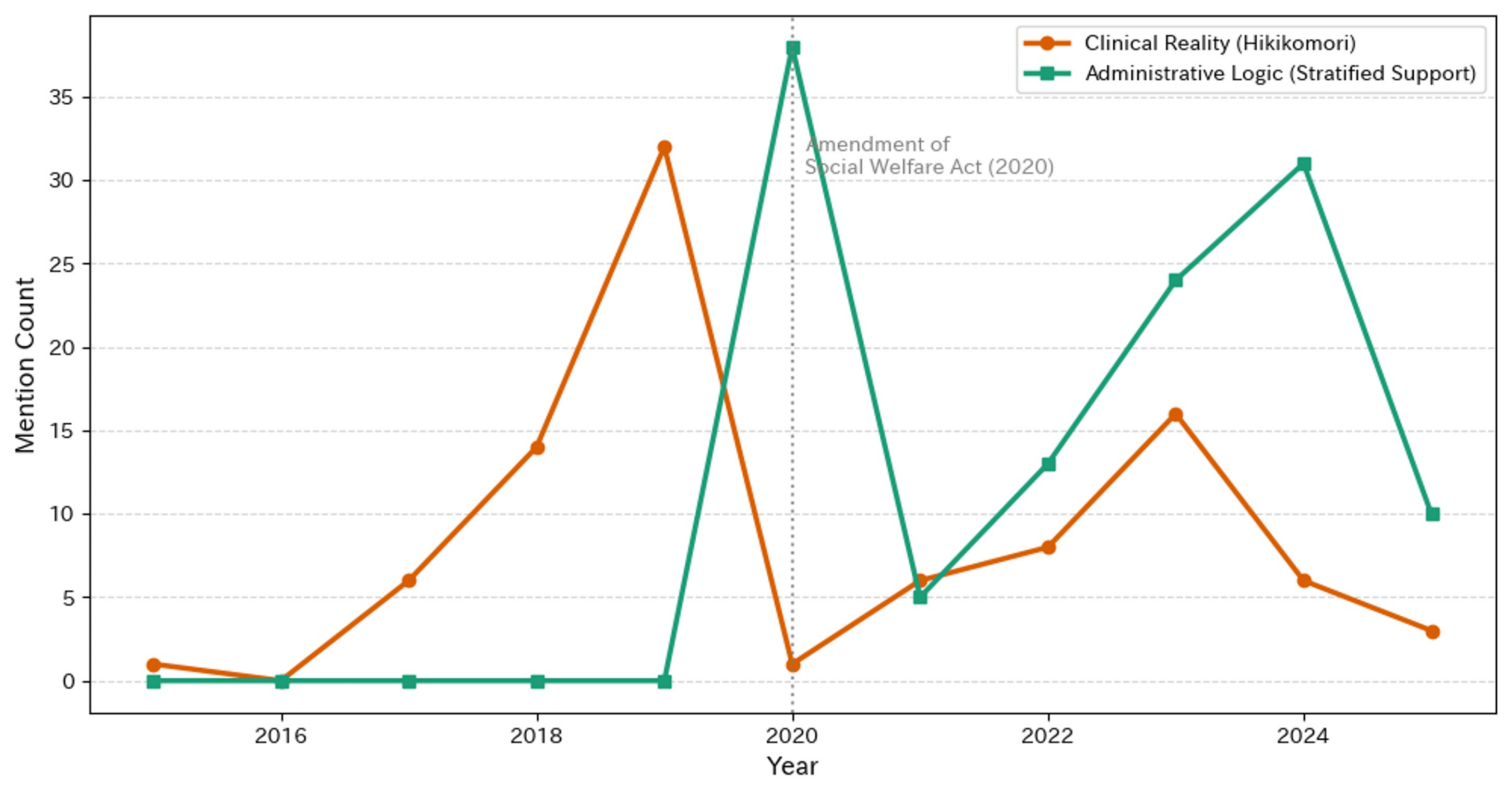
Policy Transition in the National Diet (2015-2025) This graph compares the annual frequency of parliamentary mentions for "clinical reality" (orange line and circles; keyword: *hikikomori*) versus "administrative logic" (green line and squares; keyword: multilayered support). The vertical dotted line marks the 2020 amendment of the Social Welfare Act, highlighting the crossover in terminology frequency

The paradigm shift in parliamentary language is visually illustrated in Figure 1. This time-series analysis highlights the inverse correlation between clinical and administrative terminology, focusing on the 2020 amendment to the Social Welfare Act.

As detailed in Table 1, the frequency of the keyword "hikikomori" (clinical reality) increased steadily to reach its peak in 2019, immediately before the legislative change. However, following the amendment in 2020, the use of this clinical term has decreased significantly. In stark contrast, the term "multilayered support" (administrative logic) surged in 2020. Although there have been minor fluctuations, administrative terminology has consistently outnumbered clinical references from 2020 to 2025, indicating that the clinical vocabulary was structurally displaced by administrative language.

**Table 1:** Annual Mention Counts of "Clinical Reality" and "Administrative Logic" in the National Diet (2015-2025) This table illustrates the "paradigm shift" where the focus of the discussion shifted from the specific issue (*hikikomori*) to the system (Stratified Support) around 2020

Year	Clinical Reality (*Hikikomori*/8050 Problem)	Administrative Logic (Stratified Support System)	Key Policy Events
2015	1	0	-
2016	0	0	-
2017	6	0	-
2018	14	0	Increasing social attention on "8050 problem"
2019	32	0	Peak of crisis discussion (incidents related to social isolation)
2020	1	38	Amendment of the Social Welfare Act (paradigm shift)
2021	6	5	Implementation of the new system (April 2021)
2022	8	13	-
2023	16	24	-
2024	6	31	-
2025	3	10	-

The characteristics of the new administrative discourse were examined through text mining regarding the "multilayered support system." Table 2 delineates the vocabulary used in this policy framework. The discourse is heavily weighted toward broad administrative categories and procedural terms, with "life" (*seikatsu*), "welfare" (*fukushi*), and "nursing" (*kaigo*) occupying the top three ranks. Notably, terms related to organizational maintenance, such as "system" (*seido*) and "consultation" (*soudan*), rank higher than terms reflecting the actual suffering of individuals, such as "isolation" (*koritsu*) and "poverty" (*konkyu*).

**Table 2:** Top 10 Most Frequent Nouns in "Stratified Support" Deliberations This table reveals the vocabulary of the "administrative logic." High-frequency words such as "consultation" and "cooperation" appear as frequently as the issues themselves ("isolation" and "poverty"), indicating a focus on the process

Rank	English Translation	Original Japanese	Frequency (Count)
1	Life/living	*Seikatsu* (生活)	201
2	Welfare	*Fukushi* (福祉)	167
3	Nursing/care	*Kaigo* (介護)	166
4	System/regime	*Seido* (制度)	134
5	Consultation	*Soudan* (相談)	131
6	Isolation	*Koritsu* (孤立)	126
7	Poverty/distress	*Konkyu* (困窮)	123
8	Challenge/issue	*Kadai* (課題)	111
9	Local government	*Jichitai* (自治体)	108
10	Cooperation/linkage	*Renkei* (連携)	101

Furthermore, the co-occurrence network analysis (Table 3) reveals the structural logic of debates. The term "cooperation" (*renkei*) functioned as a central hub, appearing in four of the top five co-occurrence pairs (linked with "consultation," "relation," "welfare," and "challenge"). This indicates that the parliamentary focus has prioritized building institutional connections over the direct resolution of clinical realities.

**Table 3:** Top 10 Co-occurrence Word Pairs in "Stratified Support" Deliberations This table visualizes the "structure of logic." The frequent appearance of the word "cooperation" (*renkei*) in the top pairs suggests that "building connections" has become the primary objective of the policy, potentially overshadowing the direct resolution of clinical realities

Rank	Node A (English)	Node B (English)	Original Pair (Japanese)	Co-occurrence Weight
1	Poverty	Life/living	Konkyu-Seikatsu	27
2	Consultation	Cooperation	Soudan-Renkei	26
3	Cooperation	Relation	Renkei-Kankei	25
4	Welfare	Cooperation	Fukushi-Renkei	25
5	Challenge	Cooperation	Kadai-Renkei	24
6	Life/living	Welfare	Seikatsu-Fukushi	23
7	Consultation	Local government	Soudan-Jichitai	23
8	Consultation	Relation	Soudan-Kankei	23
9	Consultation	Welfare	Soudan-Fukushi	23
10	System	Life/living	Seido-Seikatsu	23

## Discussion

The shift in language within National Diet discussions around 2020, from the raw clinical reality of "hikikomori" to the impersonal administrative phrase "multilayered support," represents a significant change in how this social withdrawal phenomenon is framed at the policy level. While such a transition can superficially appear as progress in addressing the issue, it has profound implications from a public health perspective concerning the social determinants of health (SDOH). *Hikikomori*, characterized by extreme and prolonged social withdrawal, is a severe mental health condition with clinically recognized dimensions. Studies have revealed that individuals with hikikomori often experience substantial impairments in mental health and social functioning and comorbid psychiatric disorders, such as anxiety, mood disorders, and schizophrenia [5,6]. Clinically, the experience of raw pain and isolation is a significant part of their reality, which calls for nuanced and direct recognition in policy-making.

The depersonalization or "sanitization of language" through bureaucratic terms such as "multilayered support" can dilute the urgency and specificity of the problem. Language in political discourse, especially in legislative bodies such as Japan's National Diet, strongly shapes social policies, budget allocations, and the design of support systems. According to the social determinants of health framework, political decisions and policies constitute "the causes of the causes," influencing health outcomes by determining the living environments and available resources for vulnerable populations. When legislative discourse abstracts the individual human suffering embedded in *hikikomori*, it risks formulating generic policies that may inadequately address complex and individualized needs. This "sanitization" of language suggests a potential structural risk; by fostering administrative logic over clinical reality, it could inadvertently contribute to resource allocation or support mechanisms that do not fully reach the most affected individuals or the frontline workers equipped to assist them. The gap between clinical reality and administrative language could undermine effective intervention strategies, since the recognition of *hikikomori* as a detailed clinical syndrome impacts the structuring of intervention programs, prioritization of funds, and tailoring of health services [7].

In contrast, policies framed solely in administrative terms may prioritize system management over individualized care. Furthermore, the literature underscores that *hikikomori's* social and psychological complexities require precise and empathetic addressing to improve health outcomes and social reintegration [8]. The clinical perspective, emphasizing "raw pain," recognizes the mental health burden and the urgent need for tailored medical and psychosocial interventions [5]. By overlooking this in favor of impersonal terminology, policies may fail to mitigate risks such as worsening mental health, social isolation, and suicidal behavior correlated with *hikikomori* [9]. The shift in the National Diet discourse around 2020 reflects a move from acknowledging the direct, lived experiences of *hikikomori* sufferers to a depersonalized, system-focused narrative. This language shift carries significant public health consequences because legislative language guides policy directions and resource allocations that fundamentally shape the social determinants affecting those with *hikikomori* and the frontline workers supporting them. Recognizing and addressing the risks of this "sanitization" are critical to developing more effective and compassionate policies that truly respond to the clinical realities underlying social withdrawal and its broader public health impact.

Discussions of community issues in Japan's Diet illustrate a critical disconnect between national policy-making rationales and the lived realities experienced by frontline specialists tasked with executing these policies. This disconnect is exemplified by goal displacement, where organizational maintenance and institutional processes overshadow the original mission of providing comprehensive support to residents. At the national level, debates increasingly emphasize the institutional logic of committee structures, budget allocations, and coordination mechanisms. This structural framing risks abstracting the policy discourse away from the urgent clinical realities faced by those in need. Frontline staff and professionals working within local governments and community settings bear the brunt of this policy-reality gap. These workers face the compounded pressure of managing increasingly complex administrative systems without the benefit of concrete, actionable solutions emerging from national debates. Consequently, they experience increased workload, bureaucratic obstacles, and inadequate institutional support, which can contribute to stress, burnout, and diminished self-efficacy.

Studies from diverse international contexts corroborate the challenges faced by frontline workers. For example, frontline healthcare and social care workers during the COVID-19 pandemic reported feelings of insufficient organizational support despite heightened demands, leading to frustration and burnout [10,11]. Similarly, frontline workers in Brazil experienced erosion of their roles and autonomy due to ambiguous and top-down policy directives, undermining their capacity to respond effectively to community health needs [12,13]. This highlights how policy decisions detached from frontline realities disempower the essential workers. Moreover, frontline workers often do not receive adequate psychosocial support or resources that are responsive to their complex and evolving challenges. Research suggests that flexible, multidimensional support systems co-designed with frontline staff input are needed to address structural, systemic, and individual barriers [11]. This includes recognizing the heterogeneity among frontline roles, from community health workers to supervisors, and providing tailored managerial and peer support that fosters affective commitment and reduces turnover intentions [14]. Examining community issues discussed in the Diet must extend beyond policy rhetoric to illuminate the structural difficulties faced by frontline healthcare specialists. Such an examination is vital to making visible the disconnect between administrative logic and clinical reality, ensuring that national decisions are grounded in the authentic conditions confronting frontline workers, thereby enabling policies that effectively support both professionals and the communities they serve.

The phenomenon described, where references to "pain" and "the faces of those affected" in political debates are replaced with bureaucratic terms such as "systems" and "coordination," leads to a shift in how social determinants of health (SDOH) are framed and acted upon in the political discourse. When political discourse substitutes the lived experiences of suffering and need with abstract administrative language, there is a risk that budget allocations might inadvertently prioritize administrative functions, such as "establishing committees" and "drawing up plans," over direct, tangible support such as frontline staffing or assistance for those experiencing poverty and isolation. This shift results in what can be called a "fallacy of composition"; while the healthcare or social support system may outwardly appear improved through increased administrative activities, the fundamental issues of isolation and poverty that constitute the true social determinants of health remain unaddressed. Consequently, the authority to define what counts as social determinants of health is appropriated from the voices of those directly affected and transferred to administrative logic, which focuses on systems rather than individual needs [15-17]. This redefinition alters the political space and the power dynamics that shape health outcomes. The disappearance of the "issue (*hikikomori*)" in discourse, replaced by increasing references to the "system (multilayered support)," exemplifies this appropriation, both visually and rhetorically. It signals a moment when the political authority to define social determinants, and thus direct health policies, displaces the direct voices and needs of affected populations with administrative frameworks shaped more by governance and bureaucracy than by lived realities. This can diminish the responsiveness of policies to actual social suffering and widen the gap between formal system improvements and the persistent health inequities experienced by individuals [15-17].

Such a discursive and policy shift aligns with broader critiques of the political management of social determinants of health. Political determinants of health, meaning the distribution of power and resources through government action or inaction and policy, form the structural roots of social determinants. When political discourse centers on administrative processes rather than the direct needs of affected populations, it can reinforce health inequities by shifting attention away from material conditions such as poverty, isolation, and social exclusion to managerial processes [17]. Moreover, this shift may reflect the tendency to depoliticize or technocratize social issues, focusing on efficiency, coordination, and systematization rather than substantive structural change or direct support [18]. Therefore, while a multilayered support system could theoretically improve the coordination of services, the absence of reference to the direct lived experiences of pain and social need risks masking the continuing reality of poverty and isolation. Without deliberate attention to these human dimensions, policies risk perpetuating inequities by prioritizing administrative goals over meaningful improvements in social determinants. This results in a "fallacy of composition," where aggregate system metrics improve but individual suffering and social determinants remain inadequately addressed, undermining real progress in public health and social welfare [15-17]. In the context of Japanese welfare policy, the terms "cooperation" and "consultation" appear with disproportionately high frequency relative to outcome-oriented words such as "solution" and "recovery," indicating a shift in emphasis from tangible assistance to the administrative process of collaboration.

For ordinary citizens, the notion of "cooperation" entails specialists from multiple disciplines coming together to directly provide assistance to those in need of help. However, within Japan's administrative apparatus, the repeated invocation of "cooperation" can mask less favorable dynamics such as "diffusion of responsibility" or "passing the buck," transforming a means of support into an end in itself. This mirrors broader tendencies in bureaucratic systems, where procedural formalities potentially hinder rather than help effective intervention. The use of co-occurrence network analysis within the National Diet's discussions illustrates this phenomenon concretely. The analytical findings show that conversations skewed toward the "how to cooperate" process, suggesting that the creation and maintenance of cooperative frameworks have become focal points rather than instruments to achieve welfare objectives. There is a concern that such a procedural focus may contribute to frontline worker exhaustion, as critical emergency responses risk being delayed by mandatory coordination meetings and bureaucratic requirements.

Consequently, the bureaucracy prioritizes formalized cooperation rhetoric, leading politicians and officials to feel that they have acted affirmatively simply by committing to "strengthen cooperation." Ironically, this rhetoric burdens field workers with increasing coordination and administrative tasks to maintain cooperation, detracting from direct engagement with individuals needing timely help. The delay and emphasis on procedural steps erode the immediacy of welfare outreach, the essential, practical support for vulnerable populations, which is the true goal of the welfare system. The welfare regime in Japan is often characterized as a hybrid system blending elements from Western welfare models; however, it exhibits distinctive features, such as a strong bureaucratic influence that can sometimes obstruct effective delivery [19]. Furthermore, cultural dimensions dictate how such frameworks operate; collectivist societies such as Japan tend to emphasize state-led welfare programs over informal charitable initiatives, fundamentally defining how "cooperation" is institutionalized in public health contexts [20]. Overall, the "goal displacement" through bureaucratic overemphasis on cooperation procedures not only impedes prompt crisis response but also results in frontline workers' burnout, undermining the welfare system's foundational purpose of providing timely and effective aid to citizens in need. This case exemplifies the complex challenges of balancing administrative coordination with practical implementation in social policy frameworks.

The analysis of parliamentary deliberations has depended on manual text analysis, with researchers painstakingly reading massive volumes of session transcripts. Owing to the physical limitations of human processing ability, much prior research has been constrained to limited samples, such as extracting only specific committees or several years of debates. Consequently, these studies have constantly faced criticism during peer review for insufficient sample sizes or the arbitrary selection of convenient data. There are countless "unpublished studies" brimming with sharp qualitative insights rooted in on-the-ground perceptions, which nevertheless disappeared from academic discourse because they failed to demonstrate statistical robustness. This is not merely a failing of individual studies but a structural tragedy brought about by analog analysis methods. This study's comprehensive analysis, utilizing APIs, attempts a technological breakthrough against the bias that traditional research has unavoidably harbored. By targeting all statement data from the past 10 years, we have made it possible to approach the "entire population itself" rather than relying on partial sampling. This resolves, at the root, the issue of bias that qualitative research has long faced and enables a quantitative analysis of the transition from the "8050 problem" to the Multilayered Support System Development Project.

The usefulness of API-based full-population surveys lies not only in eliminating bias. They are also extremely effective in guaranteeing "reproducibility," which is the most highly valued aspect of scientific processes. In conventional manual qualitative analysis, there has always been the risk that different researchers would reach different conclusions from the same transcripts due to subjective interpretation or political standpoint, an arbitrariness of interpretation that could not be excluded. However, the morphological analysis and co-occurrence network analysis with Python (Python Software Foundation, Wilmington, DE) code utilized in this study will always produce the same network diagrams and figures from the same dataset, regardless of who runs the analysis or when. For social issues such as the 8050 problem, prone to emotional conflict, the fact that the use of the term "hikikomori" (social withdrawal) decreased while "coordination" increased must be presented as an unshakable "mathematical truth," not merely as the researcher's "impression." This method provides an objective foundation, equivalent to that in the natural sciences, for policy analysis and social epidemiological studies, which have until now been regarded as low in evidential value. Furthermore, the full-population approach is also powerful in making visible the "absences," the things that were not spoken, which humans often fail to notice. While people can focus on "discussions that exist," it is difficult to recognize "words that ought to have been present but have disappeared."

In the time-series data of this study (Table 1), the fact that the term "hikikomori" virtually disappeared to the level of statistical noise after 2020 is a phenomenon that cannot be captured through partial sampling. Because we took a bird's-eye view of the entire population, we were able to detect the "silent fact" that the very existence of the people concerned had vanished from the discussion arena at the policy turning point. In this sense, the method suggests its potential as a powerful public health tool for monitoring how SDOH may be rendered politically invisible.

Limitations

This study has limitations in terms of translating research findings into policy actions. While the API-based quantitative analysis successfully visualized the semantic substitution of clinical reality with administrative logic, the scope of this study is strictly limited to the dissemination of these analytical results in academic literature. I identified a critical disconnect in the policy formation process, where the lived experiences of vulnerable populations are obscured by procedural terminology. However, this research lacks the instrumental capacity to directly intervene in or rectify legislative discourse. The "feedback loop" proposed in the conclusion relies entirely on the receptivity of policy-makers, and there is no guarantee that providing objective evidence of this "depersonalization" will reverse the bureaucratic inertia that prioritizes system maintenance over individual clinical needs. Therefore, the ultimate limitation lies in the structural gap between "diagnosing" policy distortion through data science and the political agency required to implement the necessary corrections.

## Conclusions

In the current landscape of open data, the use of public APIs provides a robust methodology for monitoring policy discourse. As demonstrated in this study, comprehensive text analysis can mitigate sampling bias and objectively visualize semantic shifts in legislative framing. Although textual analysis alone cannot establish direct causal links to real-world public health outcomes, it serves as an essential tool for identifying how social issues are politically constructed. The development of such data infrastructure enables researchers and citizens to scrutinize whether the policy language aligns with clinical realities. Establishing a feedback loop based on objective discourse analysis may contribute to more responsive policy-making. Ultimately, maintaining a critical awareness of how the social determinants of health (SDOH) are framed is a vital step toward developing support systems that truly reflect the needs of vulnerable populations.

## Appendices

Detailed methodological parameters for text analysis

Data Collection Parameters (NDL API)

Endpoint: National Diet Library API (https://kokkai.ndl.go.jp/api/speech).

Timeframe: January 1, 2015, to December 31, 2025.

Data format: JSON (recordPacking: "json").

Target keywords: Exact matches for "hikikomori" and "Project for Developing a Stratified Support System."

Morphological Analysis and Token Filtering Rules

Text segmentation and part-of-speech (POS) tagging were executed using the Python-based morphological analyzer "Janome." To extract meaningful policy issues and exclude grammatical noise, the following strict token-filtering rules were applied: POS inclusion and length constraint.

POS inclusion: Only tokens classified as "nouns" and "verbs" were extracted. Specifically, the extraction was limited to independent words, general nouns, and verbal nouns.

Length constraint: Single-character tokens (e.g., length = 1) were systematically excluded to eliminate the contextual noise.

Stop-Word Lists

To prevent highly frequent but semantically empty words from skewing the analysis, a custom stop-word list was applied during preprocessing. This list includes three categories.

General verbs and auxiliaries: Commonly used verbs lacking specific policy meanings (e.g., *suru* {do}, *iru* {be}, *omou* {think}, and *kangaeru* {consider}).

Parliamentary jargon: Standard procedural terms used in the Diet that do not reflect policy content (e.g., *Iin* {committee member}, *daijin* {minister}, *shitsumon* {question}, and *touben* {answer}).

Search term components: To prevent the target keyword itself from artificially dominating the frequency counts, the constituent terms of the administrative system (e.g., *juso* {stratified}, *shien* {support}, *taisei* {system}, *seibi* {development}, and *jigyo* {project}) were removed prior to counting.

Co-occurrence Network Settings

For the semantic structure analysis, co-occurrence relationships were calculated based on sentences within the same speech. Edges were drawn only for word pairs exhibiting a Jaccard coefficient of 0.1 or higher, and the visualization was restricted to the top 60 most strongly connected nodes to ensure interpretability.
